# Visual acuity in larval zebrafish: behavior and histology

**DOI:** 10.1186/1742-9994-7-8

**Published:** 2010-03-01

**Authors:** Marion F Haug, Oliver Biehlmaier, Kaspar P Mueller, Stephan CF Neuhauss

**Affiliations:** 1 Institute of Molecular Life Sciences, University of Zurich, Winterthurerstrasse 190, CH-8057 Zurich, Switzerland; 2 Light Microscopy Centre, Swiss Federal Institute of Technology (ETH), Schafmattstrasse 18, CH - 8093 Zürich, Switzerland

## Abstract

**Background:**

Visual acuity, the ability of the visual system to distinguish two separate objects at a given angular distance, is influenced by the optical and neuronal properties of the visual system. Although many factors may contribute, the ultimate limit is photoreceptor spacing. In general, at least one unstimulated photoreceptor flanked by two stimulated ones is needed to perceive two objects as separate. This critical interval is also referred to as the Nyquist frequency and is according to the Shannon sampling theorem the highest spatial frequency where a pattern can be faithfully transmitted. We measured visual acuity in a behavioral experiment and compared the data to the physical limit given by photoreceptor spacing in zebrafish larvae.

**Results:**

We determined visual acuity by using the optokinetic response (OKR), reflexive eye movements in response to whole field movements of the visual scene. By altering the spatial frequency we determined the visual acuity at approximately 0.16 cycles/degree (cpd) (minimum separable angle = 3.1°). On histological sections we measured the retinal magnification factor and the distance between double cones, that are thought to mediate motion perception. These measurements set the physical limit at 0.24 cpd (2.1°).

**Conclusion:**

The maximal spatial information as limited by photoreceptor spacing can not be fully utilized in a motion dependent visual behavior, arguing that the larval zebrafish visual system has not matured enough to optimally translate visual information into behavior. Nevertheless behavioral acuity is remarkable close to its maximal value, given the immature state of young zebrafish larvae.

## Background

The visual system extracts optical information from the environment in order to adjust behaviors. One of the core properties of any visual system is the ability to distinguish between two objects at a given angular distance, referred to as visual acuity.

This limit of spatial resolution can be influenced by many properties of the visual system, including the optics of the eye, accommodation, neural properties of the retina and visual processing in higher brain centers. The ultimate physical limit is given by the density of photoreceptors in the retina.

The relationship between photoreceptor density and visual acuity was first discussed in the 19^th ^century by Bergmann and Helmholtz [1,2]. They assumed that to perceive two visual stimuli as separate, at least one unstimulated photoreceptor must lie in between two activated receptors. Hence the critical frequency, also referred to as Nyquist frequency, at which the two separate stimuli can be resolved, should not exceed 1/2 s, where s stands for photoreceptor spacing [3]. Below his frequency the image is undersampled and distorted, a phenomenon called spatial aliasing.

Interestingly the observer's criteria for resolving a grating might be less strict than the criteria defined by Bergmann and Helmholtz. Humans, for instance, can reach a higher visual acuity than would be expected from their photoreceptor spacing alone, presumably by sampling information from slightly divergent images generated by eye and head movements [4].

In order to reach the upper limit of visual acuity as stated by the Shannon theorem using the Nyquist frequency, all other factors impinging on image quality must be near optimal. Among these factors is the quality of the optical apparatus of the eye (cornea, lens) and its ability to focus the image onto the receiving photoreceptor cells. Such an eye is called emmetropic. Additionally, the neural mechanisms of receiving and processing the visual information might also limit spatial resolution. Teleost (ray-finned) fishes are good experimental models to study the relationship between photoreceptor spacing and visual acuity, since they are amenable to behavioral experiments and have a regular photoreceptor array [5-9]. Moreover, behavioral experiments of the adult goldfish assume that mainly cone density restrains visual acuity and that the optic apparatus is not a limiting factor [10,11].

The zebrafish retina contains a row mosaic pattern [8,12] which is composed of rows of alternating blue- and UV-sensitive single cones that alternate with double cones. The parallel rows are aligned such that the green-sensitive members of the double cones flank the short single UV-cones, and the long single blue-cones are nearer to the red-sensitive member of the double cone [13]. This pattern is established early in zebrafish development, in conjunction with the fast maturation of the visual system. By 5 dpf (days post fertilization) a number of visual behaviors can be evoked [14-16], and sum field potentials of the retina in response to light can be recorded [17].

This rapid maturation of vision and the genetic amenability has made the zebrafish visual system an attractive model to study the genetics of vertebrate vision, with many visually impaired mutant lines available [16,18-21]. In order to interpret the results of genetic and pharmacological perturbations on visual performance, it is necessary to define the limits of vision in the wild-type.

Therefore we determined the visual acuity of larval zebrafish by determining the cut-off frequency using the behavioral optokinetic response paradigm [22]. This behavioral measure for visual acuity was then related to the theoretical maximal acuity dictated by photoreceptor spacing. We found that the behaviorally defined acuity was about two fold lower than the theoretical maximum. This indicates that the young zebrafish brain can utilize a large part but not the maximal visual information content to drive a behavioral response.

## Methods

### Fish maintenance and breeding

Fish were maintained and bred as previously described [23] and kept under a 14 h/10 h light/dark cycle. The wild-type strain used for larval behavioral and histological analysis was Tübingen (Tü). Embryos were raised at 28°C in E3 medium (5 mM NaCl, 0.17 mM KCl, 0.33 mM CaCl_2 _and 0.33 mM MgSO_4_) and staged according to development in days post fertilization (dpf).

For light microscopy, embryos were treated with 0.2 mM phenylthiourea (PTU, Fluka-Sigma-Aldrich, St. Louis, Missouri, USA) from 8 to 56 hours post fertilization (hpf).

### Behavioral analysis of larval zebrafish

All measurements were conducted between 10 am and 5 pm. Optokinetic stimulation of larval fish was performed as recently described for adult fish [24] with some adaptations: As described in the publication of Rinner [22], larval fish were placed in a 35 mm Petridish containing 3% pre-warmed methylcellulose. With a contrast of 90% (contrast is normalized, such that 100% is the maximal contrast that can be achieved), sine-wave gratings with variable spatial frequencies were presented, first increasing incrementally from the lowest to the highest value followed by a decrease back to the lowest one. Each spatial frequency was presented for 9 seconds with changing direction every 3 seconds to minimize saccade frequency. Before the measurement started, larval fish were pre-stimulated with a spatial frequency of 0.06 cpd for 9 seconds. Varying spatial frequencies were presented with a constant angular velocity of 7.5 degrees per second.

Images of the larva were taken at 5 frames per second by a CCD-camera (Guppy F-038B NIR, Allied Vision Technologies, Germany) attached to a dissecting microscope. The eyes were detected by custom-made software based on LabView 7.1 and NI-IMAQ 3.7 (National Instruments, USA), determining angular position and calculating angular velocity of each eye in real time [24].

Raw measurements of eye velocities were further processed by filtering out saccadic movements with the help of following empirically tested formula: If eye velocity (*υ*) in a frame (*f*) exceeds 20 deg/sec, eye velocity of this frame, as well as of the two preceding frames, is replaced with the eye velocity 3 frames before (*υf*...*f*-2) is set to *υ *(*f*-3)), the eye velocities of the 2 following frames are replaced by the value 3 frames after (*υf*+1....*f*+2) is set to *υ *(*f*+3)). In this way, not only the saccadic peaks are filtered out, but also the curve is smoothened around saccades. After filtering saccades, the velocity curve is smoothened by a running average of 7 frames (*υ *(*f*) = Σ *υ*(*f*-3...*f*+3)/7).

To quantify visual acuity, eye velocities of wild-type zebrafish larvae were measured as a function of spatial frequency of the moving grating. Eye velocity traces of both eyes were integrated and the average velocity for each spatial frequency was calculated. Subsequently, visual acuity was calculated as the optical cut-off frequency, which is the finest grating an eye still can resolve [3]. At a spatial frequency of 0.2 cpd, the larvae clearly did not react to the stimulus anymore but showed only spontaneous eye movements. The average eye velocity measured at this spatial frequency was taken as zero-value, and all other values were statistically compared to this one with a paired one-tailed t-test. OKR-graphs, calculation of the cut-off frequency as well as statistical analysis were accomplished in Prism 5 (GraphPad Software, San Diego, USA). Graphs were generated using R 2.9.2 http://www.R-project.org.

### Histology

Larval zebrafish were fixed at the appropriate developmental stage in 4% paraformaldehyde (PFA) in 0.1 M phosphate buffer (PB; pH 7.4) for 45 minutes at room temperature (RT) and washed twice with 0.05 M PB saline (PBS; pH 7.4) for 10 minutes at RT.

For standard histology, fixed larvae and eye cups were dehydrated in a graded series of ethanol-water mixtures at room temperature for 15 minutes each. Subsequently, the tissue was embedded in resin (Technovit 7100, Kulzer, Wehrheim, Germany). Microtome sections (3-5 *μ*m) were prepared and mounted on glass slides (Menzel Gläser, Braunschweig, Germany). Sections were air dried at 60°C, stained with Richardson solution (1:1:2 1% methyl blue : 1% borax : 1% azure II in deionized water), overlaid with Entellan (Merck, Darmstadt, Germany), and coverslipped. Images were taken with a black/white camera (F-View) under an Olympus compound microscope (BX61, Olympus Optical Co. LTD, Japan).

### Immunocytochemistry

Paraformaledehyde fixed larvae were cryoprotected by overnight emersion in 30% sucrose at 4°C. Larvae were subsequently embedded in Cryomatrix (O.C.T.™, Sakura, Tissue-Tek, the Netherlands) at RT, rapidly frozen with liquid N_2 _and stored at -20°C until further use. 25 *μ*m thick tangential sections of larvae were cut with a Cryostat (Leica CM1850, Leica Microsystems GmbH, Wetzlar, Germany) at -20°C and mounted on positively charged glass slides (SuperFrost^®^Plus, Menzel Gläser). Sections were air dried at 37°C for at least 1 hour and stored at -20°C. For immunohistochemistry, the slides were thawed and washed three times with PBS at RT. To prevent unspecific binding of the antibody, a blocking solution (10% normal goat serum (Chemicon) and 1% bovine serum albumin (Fluka-Sigma-Aldrich) in 0.3% PBS/Triton X-100) was added for 1 hour at RT. Sections were incubated overnight at 4°C in the primary antibodies in blocking solution. As primary antibodies, Zpr1 (DSHB, University of Iowa, Iowa City, USA) 1:20 (raised in mice) and blue opsin (kindly obtained from Tom Vihtelic) 1:250 (polyclonal rabbit antibody) were used [25]. To obtain specific stainings and to exclude false positive results due to autofluorescence, the antibodies were first individually tested under varying conditions. Staining was visualized by using Alexa Fluor 488 anti-mouse (1:1000, Invitrogen AG, Basel, Switzerland) and Alexa Fluor 568 anti-rabbit (1:500, Invitrogen AG) as secondary antibodies. Subsequently, stained sections were coverslipped with Mowiol (polyvinyl alcohol, Fluka-Sigma-Aldrich).

Slides were viewed with a Leica confocal microscope (Leica SP2, Leica Microsystems GmbH). Confocal z-stacks were analyzed by using Imaris (Imaris 5.0.1, Bitplane AG, Zurich, Switzerland).

### Calculation of theoretical visual acuity and analysis

**Figure 1:**
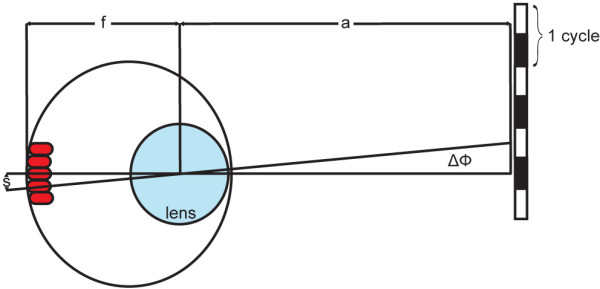
**Trigonometric calculation used for obtaining a theoretical value of the visual acuity**. Visual acuity was calculated in cycles per degree (cpd) using the following formula: 1/(2(arctan(s/f)). f: focal length; s: distance between the center of two red-green double cones; ΔΦ: inter-receptor angle at the nodal point of the lens.

After taking pictures of the obtained fluorescently labeled zebrafish sections, the center-to-center distance between two red-green double cones was measured with ImageJ software (ImageJ 1.37a, Wayne Rasband, National Institutes of Health, USA). The physical maximal cut-off frequency was received by trigonometric analysis using the red-green double cone spacing as well as the focal length of the fish eye (Fig. 1). Significance between 4, 5, and 6 day old zebrafish was ascertained in a one-way ANOVA and a Tukey's Multiple Comparison post hoc test performed in Prism 5 (GraphPad Software).

## Results

### Behavioral measurement of visual acuity

In order to experimentally measure visual acuity, we used the optokinetic reflex of zebrafish larvae. These reflexive eye movements in response to large field movement in the surround consist of a smooth pursuit movement in the direction of the moving stimulus and a fast saccadic reset. This stereotypic behavior can be robustly elicited and easily quantitatively measured [22,26]. As a measure of visual performance, we measured the gain of the smooth pursuit movement over a range of spatial frequencies using high contrast black-and-white stripes.

**Figure 2:**
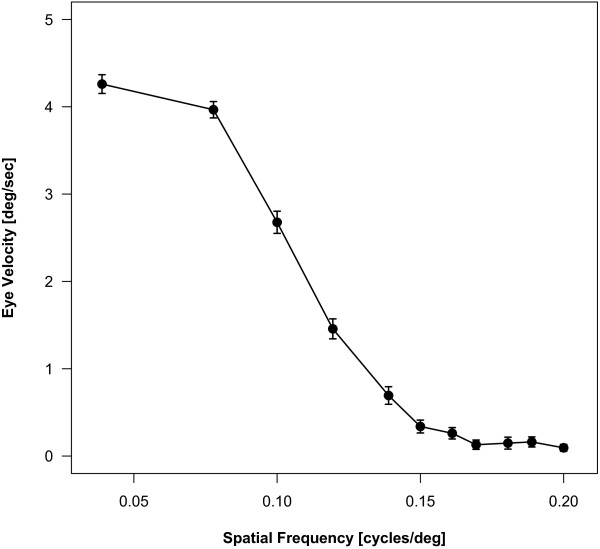
**Behavioral OKR measurements at 5 dpf**. Smooth pursuit eye velocity at 11 different spatial frequencies of the stimulation between 0.04 and 0.2 cpd was measured at a constant angular velocity of 7.5 degrees per second. The value obtained at 0.2 cpd was set as the zero value. For statistical analysis, the significance between each data point and the velocity at 0.2 cpd was calculated. Statistical analysis reveals highly significant differences between the data points of 0.04 and 0.15 cpd compared to 0.2 cpd (p < 0.001). At 0.16 cpd (p = 0.0055) the optokinetic response is still elicited, whereas at 0.17 cpd (p = 0.1557) the values do not differ significantly anymore. 0.16 cpd represents the finest grating the eye still can resolve, which is equal to the cut-off frequency or the visual acuity of the fish.

Starting with the lowest spatial frequency (0.04 cpd) we gradually reduced the width of the stripes until no consistent eye movements were elicited and subsequently decreased the spatial frequency back to starting levels. We compared larvae at 4 (n = 59), 5 (n = 74) and 6 (n = 65) dpf to reveal differences in visual behavior at these different stages (data not shown). A two-way ANOVA for repeated measurements revealed no significant dependence on age ([F (df = 2) = 2.014; p = 0.161]). Between the measured spatial frequencies of 0.14 and 0.2 cpd the fish clearly stop to move their eyes and the curve approaches a baseline value. Since most visual mutants in zebrafish are described to have defects in vision at 5 dpf, and since no age-dependent difference is observed, we concentrated our study on 5 day old larvae. We used a more specified paradigm that concentrates on the range where the cut-off frequency was expected from our previous measurements. At a given optimal contrast of 90% all zebrafish larvae (n = 41) follow the stripes best at 0.04 cpd (Fig. 2). For statistics, the value at 0.2 cpd was taken as zero value. Since the fish cannot separate the stripes anymore it elicits only spontaneous eye movements. Between 0.16 and 0.17 cpd and the difference between the graph and the baseline drops down from significant (p = 0.0055 at 0.16 cpd) to not significant anymore (p = 0.1557). Therefore, the cut-off frequency of 5 day old zebrafish is 0.16 cpd (minimum separable angle = 3.1°).

In order to exclude that the projected stripes are out of focus for the larva, we repeated the same measurements with the same stripe width at different distances (33 and 62 mm versus 45 mm).

We measured no significant difference in visual acuity, suggesting that stimuli under all three conditions are in focus (data not shown).

### Photoreceptor spacing as a physical limit of visual acuity

According to the Shannon sampling theorem, the highest spatial frequency at which a pattern can be faithfully transmitted to higher visual centers is half the spatial frequency of the photoreceptor mosaic, also known as the Nyquist frequency [3]. Therefore, we set out to measure how closely the behaviorally determined visual acuity matches the physical limit imposed by photoreceptor spacing. The contribution of rod photoreceptors can be neglected, since at 5 dpf they contribute little to larval vision. Furthermore, our experiments operate well in the photopic range, where rod photoreceptors would be driven into saturation.

In fish there is good evidence that the relevant photoreceptors for motion detection are the red-green double cones [27-29]. Therefore we determined the mean distance between these cone subtypes to calculate the resolution limit imposed by photoreceptor spacing.

We prepared tangential section with vertically sliced photoreceptors, which allowed us a top view onto the cone mosaic. Since the cone mosaic is established at young larval stages [12,30], we quantified the distance between a double cone and a blue cone and calculated, based on this value, the distance between two double cones.

**Figure 3:**
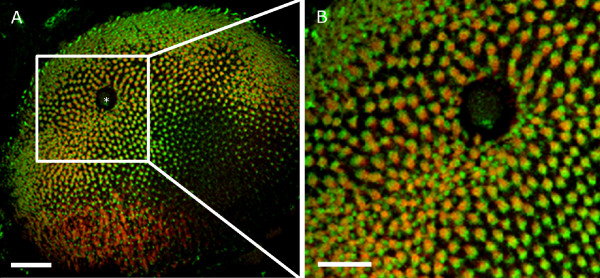
**Confocal image of an immunohistochemically labeled 6 dpf zebrafish retina**. Red-green double cones and blue cones were each labeled with a specific primary antibody and marked with a secondary antibody containing a fluorescent tag. Double cones are stained in red, blue cones in green. To obtain accurate values for the calculation of visual acuity, the center-to-center distance between two red-green double cones was measured. A: The asterisk depicts the exit of the optic nerve. B: Magnification of the cutout of C revealing the cone mosaic of the zebrafish retina. Scale bar in A = 40 *μ*m, scale bar in B = 10 *μ*m.

In larval zebrafish, light micrograph sections already revealed an even distribution of photoreceptors in the retina and the distinct retinal layers can be easily discerned (data not shown). In order to facilitate an unequivocal identification of different cone types, we labeled red-green double cones and blue sensitive short single cones with specific antibodies (Fig. 3). 307 measurements of 12 different eye cups were performed between randomly chosen red-green double cones and blue cones. The average distance between double cones and blue cones is 2.54 *μ*m ± 0.02 *μ*m at 5 dpf (this and all following errors are given as SEM). The low variation of these distances attests to the regularity of the cone mosaic already at this young larval stage.

We performed the same analysis at 4 dpf and 6 dpf which resulted in comparable distances (2.48 *μ*m ± 0.02 *μ*m at 4 dpf; 2.47 *μ*m ± 0.02 *μ*m at 6 dpf). Statistical analysis revealed no differences for the obtained distances between 4, 5, and 6 dpf (p > 0.5). We also performed a similar analysis in the adult retina. Since the adult mosaic is more regular, there are two measurable distances between red-green double cones: within or between a row of photoreceptors. To obtain the shortest distance between two red-green double cones, we considered the distance within a row of red-green double cones. The calculated mean distance between a blue and a double cone in the adult retina is 4.75 *μ*m ± 0.03 *μ*m.

In order to transform absolute retinal distances to degrees of visual angle, the retinal magnification factor (retinal distance per degree visual angle) needs to be taken into account. Therefore, we determined the distance between the nodal point of the lens and the photoreceptor outer segments at 139.2 *μ*m ± 1.13 *μ*m (n = 34) and the lens radius at 50.7 +/- 0.62 *μ*m (n = 34) on transverse larval sections. With this measure we can calculate the magnification factor and determine the separation of the relevant double cones. For the 5 dpf retina we obtained a value of 2.09 degree. According to the sampling theorem, this is the theoretical minimum separable angle. In line with the behavioral data, we see no significant difference between the analyzed larval stages.

For comparison we subjected the adult retina to a similar analysis. We measured a lens to photoreceptor distance of 949.1 *μ*m ± 25.55 *μ*m (n = 10) and a lens radius of 433.8 *μ*m ± 14.64 *μ*m (n = 10), calculating to a maximal visual acuity of 0.871 cpd or a minimum separable angle of 0.57 degree

## Discussion

In this study we compared the behaviorally measured visual acuity in zebrafish larvae with the theoretical maximum as confined by photoreceptor spacing. Visual acuity was experimentally determined by measuring the gain of the optokinetic reflex (OKR) evoked by moving stimuli of varying spatial frequency. We found that the experimentally determined acuity is close to but does not quite reach its physical limit. Visual acuity of 5 day old larval zebrafish was determined to be 0.16 cpd, or about 3.1 degrees. Morphological measurements based on photoreceptor distance led to a (theoretical) cut-off frequency of 0.24 cpd or 2.09 degrees.

We measured visual acuity of zebrafish, aiming for conditions where acuity is not limited by contrast or illumination. We also changed the viewing distance and measured no difference in visual behavior after adjusting to reach the same subtended angle, suggesting that under all conditions the stimulus is in focus. Nevertheless, we can not completely exclude that a feature of our set-up limits the efficacy of this behavior. For instance eye speed might be slightly slowed down by the embedding medium that would result in a diminished gain.

In this context it is useful to compare behavioral estimates of visual acuity obtained with other behavioral paradigms. One study investigated the effect of abnormal lighting during ontogeny on zebrafish optomotor behavior [31]. Although this study was done on slightly older larvae (7-9 dpf), the measured acuity of 0.175 cpd is well in the range of our estimate.

A number of biological and physical factors could constrain visual system performance. Acquisition and processing of information in the larval retina may be suboptimal. At 5 dpf, the retina is remarkably well developed, e.g. with photoreceptor synapses that show all signs of pre- and postsynaptic specializations, including signs of morphological plasticity. However, synaptic maturation is not fully completed at that stage, indicated by the lower number of ribbons and spinules per synapse as compared to the adult retina [32]. How a smaller number of spinules and ribbons effect visual acuity is unknown, but likely marginal in impact.

Fish have a large pupillary opening in comparison to their focal length, which is optimized for light-gathering capability but leads to considerable chromatic aberration. However, the lens, which is built of concentric layers with different refraction indices (multifocal lens), allows improved spatial resolution at the cost of image contrast [33].

The zebrafish eye becomes emmetropic at about 3 dpf [14] and extraocular muscles appear adult like at that stage [34]. Hence focus and the effector muscles are unlikely to be limiting.

A previous study reported that the optokinetic response of zebrafish larvae differs depending on the direction of the stimulus [35]. The response of temporal-to-nasal presented stimuli is higher at low spatial frequencies, however, it is reversed with an increasing spatial frequency. In our study we averaged values of both eyes and both stimulus directions. Hence, a separation of stimulus directions might lead to a higher visual acuity in one direction. However, since fish spot their surrounding with both eyes and in every direction in their natural habitat, we deemed the results biologically more relevant, when averaged over all combinations.

Finally, it is difficult to estimate the efficacy of visual processing in higher visual centers of the brain, as is the processing into motor commands in the brain. It is likely that many of the relevant neural systems are immature and may improve during further development.

Studies of visual acuity during the ontogeny of the striped triplefin showed that behavioral acuity was much poorer than histological estimates [36]. However this discrepancy may be partially explained by the larval myopia that can not serve as an explanation for larval zebrafish [14].

Our histological estimates of visual acuity was aided by the photoreceptor mosaic that is already apparent at larval stages, albeit less regular than in the adult retina. We based our histological estimate of acuity on the distance of double cones. There is good experimental evidence that motion detection, as assessed by our behavioral paradigm, is mediated by modulation of the red and green sensitive double cones [27,28]. In our experiments UV-cones and rod photoreceptors should anyway not influence the behavior, since the projector does not emit UV-light and the few potentially functional rod photoreceptors are well in saturation under the given brightness.

In summary, although our behavioral estimates of visual acuity do not quite reach the maximal acuity estimated from photoreceptor distance, it is nevertheless remarkable close. Why the theoretical maximal acuity is not reached is currently unknown, but likely a combination of various biological and physical factors contribute. It will be interesting to make a similar comparison of morphological and behavioral acuity in adult fish. Such an analysis will approach an answer to the question if maturing neuronal circuits are truly limiting.

## Conclusion

Acuity, the ability to distinguish two objects as separate, is a key feature of any visual system. A comparison of the maximal acuity confined by photoreceptor spacing with actual behavioral measurements in the larval zebrafish reveals a close but not perfect match. This indicates that the larval visual system can not fully translate visual information into behavior. However, the experimentally determined acuity is still remarkably close, given the developmental stage of the larvae. This affirms the remarkable capabilities of the zebrafish visual system at that stage. Since young larvae are routinely used for genetic and pharmacological perturbations of vision, this study provides a valuable reference value for normal visual acuity.

## Competing interests

The authors declare that they have no competing interests.

## Authors' contributions

MFH conducted the research, KPM contributed to the behavioral experiments, OB helped in histological analysis, MH and SCFN drafted and wrote the manuscript, SCFN planned and coordinated the study. All authors edited and approved of the manuscript.
